# Enamel matrix derivative as adjunctive to non-surgical periodontal therapy: a systematic review and meta-analysis of randomized controlled trials

**DOI:** 10.1007/s00784-022-04474-1

**Published:** 2022-04-07

**Authors:** Andrea Roccuzzo, Jean-Claude Imber, Alexandra Stähli, Dimitrios Kloukos, Giovanni E. Salvi, Anton Sculean

**Affiliations:** 1 Department of Periodontology, School of Dental Medicine, University of Bern, Freiburgstrasse 7, CH-3010 Bern, Switzerland; 2 Robert K. Schenk Laboratory of Oral Histology, School of Dental Medicine, University of Bern, Bern, Switzerland; 3 Department of Orthodontics and Dentofacial Orthopedics, School of Dental Medicine, University of Bern, Bern, Switzerland

**Keywords:** Periodontitis, Enamel matrix derivate, Periodontal pockets, Non-surgical periodontal therapy, Scaling and root planing

## Abstract

**Objectives:**

To assess the potential additional benefit of the local application of enamel matrix derivative (EMD) on the clinical outcomes following non-surgical periodontal therapy (NSPT) (steps 1 and 2 periodontal therapy).

**Materials and Methods:**

A systematic literature search was performed in several electronic databases, including Medline/PubMed, Embase, The Cochrane Register of Central Trials (CENTRAL), LILACS, and grey literature. Only randomized controlled clinical trials (RCTs) were eligible for inclusion. Clinical attachment level (CAL) change (primary outcome), probing pocket depth (PPD), and bleeding on probing (BoP) reductions (secondary outcomes) were evaluated. The Cochrane Risk of Bias tool (RoB 2.0) was used to assess the quality of the included trials. Weighted mean differences (WMDs) and 95% confidence intervals (CIs) between test and control sites were estimated using a random-effect model for amount of mean CAL and PPD change.

**Results:**

Six RCTs were included for the qualitative analysis, while data from 4 studies were used for meta-analysis. Overall analysis of CAL gain (3 studies) and PPD reduction (4 studies) presented WMD of 0.14 mm (*p* = 0.74; CI 95% − 0.66; 0.94) and 0.46 mm (*p* = 0.25; CI 95% − 0.33; 1.26) in favor of NSPT + EMD compared to NSPT alone respectively. Statistical heterogeneity was found to be high in both cases (*I*^2^ = 79% and 87%, respectively).

**Conclusions:**

Within their limitations, the present data indicate that the local application of EMD does not lead to additional clinical benefits after 3 to 12 months when used as an adjunctive to NSPT. However, due to the high heterogeneity among the studies, additional well-designed RCTs are needed to provide further evidence on this clinical indication for the use of EMD.

**Clinical relevance:**

The adjunctive use of EMD to NSPT does not seem to additionally improve the clinical outcomes obtained with NSPT alone.

**Supplementary Information:**

The online version contains supplementary material available at 10.1007/s00784-022-04474-1.

## Introduction

Periodontitis is a chronic disease caused by bacterial biofilm which, when left untreated, leads to the destruction of the tooth-supporting apparatus ultimately leading to tooth loss [1–7]. The pathogenesis of periodontitis is driven by the complex host-biofilm interactions that result in the dysbiosis of the microbiome and the dysregulation of the host immune response [1–4, 6, 7].

Non-surgical periodontal therapy (NSPT) aims to remove supra- and subgingival hard and soft bacterial deposits and has been proven to be clinically effective by leading to substantial clinical improvements evidenced by clinical attachment level (CAL) gain and probing pocket depth (PPD) reduction [8]. Nevertheless, challenging anatomical sites with impaired access, such as furcation areas [9] or deep periodontal pockets [10], are difficult to be accessed and may further serve as shelter for residual subgingival calculus and bacterial deposits. This will in turn maintain the inflammation and lead to further loss of periodontal supporting tissues [11].

Various surgical procedures have been shown to be effective in additionally reducing the residual pockets persisting after NSPT and, in certain situations, to even effectively restore intrabony [12, 13] and class II furcation defects [14, 15].

Since its introduction more than 25 years ago, enamel matrix derivatives (EMD) have advanced to be generally accepted as a standard in regenerative periodontal therapy [16, 17]. Animal and human histological studies [16, 18, 19] along with numerous clinical trials have proven the ability of surgical periodontal therapy in conjunction with EMD to facilitate periodontal wound healing/regeneration and enhance the short- and long-term clinical outcomes [17]. Particularly in deep intrabony defects, open flap debridement (OFD) + EMD has shown superior results compared to OFD alone [14, 15].

A number of clinical studies have also evaluated the effect of EMD used in conjunction with OFD in periodontal pockets without an intrabony component (i.e., in so-called suprabony defects). The results revealed greater CAL gains following the additional use of EMD compared with the use of OFD alone [20–24]. This has been very recently also corroborated by a randomized clinical trial (RCT) with a 12-month follow-up [22]. The authors reported a significant benefit of OFD + EMD over OFD alone in terms of CAL gain (3.4 ± 0.6 mm vs 1.8 ± 0.6 mm), decrease in probing pocket depth (PPD) (3.9 ± 0.6 mm vs 3.2 ± 0.6 mm), and increase in gingival recession (0.5 ± 0.7 mm vs 1.4 ± 1.0 mm) favoring the use of EMD [22].

The effects of EMD are multifaceted and have been thoroughly investigated over the last two decades [17]; apart from its anti-inflammatory properties, it has been reported that EMD positively influence wound healing, prevent or retard epithelial cell migration, promote angiogenesis, and enhance proliferation of periodontal and osteoprogenitor cells and of fibroblasts [17].

When EMD were used in conjunction with NSPT, earlier studies have failed to reveal additional PPD reduction [25, 26]. However, Wennström and Lindhe [26] observed a greater reduction of gingival inflammation (i.e., less bleeding on probing) following the use of EMD in conjunction with NSPT compared to the control group (i.e., SRP alone) after 3 weeks. More recently, 3 RCTs comparing NSPT with or without subgingival application of EMD have shown some additional clinical improvements following the use of EMD as compared to the control NSPT alone [27–29]. Even though the use of EMD in conjunction with NSPT appears to offer some interesting perspectives for the clinician, at present it is still unclear to what extent this new clinical indication is also supported by data from RCTs. According to the best of our knowledge, at present no systematic review has assessed the literature and pooled the effect estimates on the additional use of EMD to NSPT. Therefore, the aim of the present systematic review was to assess the potential additional benefit of the local application of EMD on the clinical outcomes following NSPT.

## Materials and methods

### Registration of the study protocol

The study protocol was submitted to the PROSPERO international prospective register of systematic reviews hosted by the National Institute for Health Research (NIHR), University of York, UK, Center for Reviews and Dissemination and was allocated the identification number CRD42021258154.

### Reporting format

The Preferred Reporting Items for Systematic Reviews and Meta-Analyses (PRISMA) were adopted throughout the process of the present systematic review [30].

### Population (P), Intervention (I), Comparison (C), Outcomes (O), and Study Design (S) (PICOS)

#### Population

Systemically healthy patients diagnosed with periodontitis grades II–IV and stage B/C [31]

#### Intervention

NSPT with single adjunctive delivery of EMD.

#### Comparison

NSPT with single adjunctive delivery of placebo or without local delivery of EMD.

#### Outcomes variables

**Primary:**Change in clinical attachment level (CAL).

**Secondary:**Change in pocket probing depth (PPD), change in bleeding on probing (BoP), residual PPD, pocket closure (i.e., PPD ≤ 3 mm or PPD ≤ 4 mm without BoP), plaque index (PI).

Patient-related outcome measures (PROMs) such as pain, satisfaction, discomfort, quality of life indicators, and economic factors.

#### Study design

Randomized controlled trials (RCTs) with either split-mouth or parallel-arm designs were considered eligible for inclusion.

### Focused questions

The following focused questions were adapted using the PICOS criteria [32]:

- In patients with untreated periodontitis, does single delivery of EMD provide adjunctive effects on CAL change compared with non-surgical mechanical instrumentation alone?
- In patients with untreated periodontitis, does single delivery of EMD provide adjunctive effects on PPD change compared with non-surgical mechanical instrumentation alone?

### Inclusion criteria

The following inclusion criteria were applied:

- Patients of any age
- Patients diagnosed with periodontitis (any case definition accepted) [31]
- Patients with untreated periodontal conditions that refer to this review’s focused questions
- Follow-up ≥ 3 months
-  ≥ 5 patients per treatment arm at follow-up
-  ≥ 5 patients at follow-up for studies with split-mouth design
- Clinical examination at follow-up
- RCTs

### Exclusion criteria

The following exclusion criteria were applied:

- Studies including patients with systemic disorders affecting periodontal therapy
- Pre-clinical studies
- Abstracts
- Letters to editors
- Narrative reviews
- Case reports
- Insufficient/unclear information not allowing data extraction
- No author response to inquiry e-mail for data clarification

### Search strategy

Detailed search strategies were developed and appropriately revised for each database, considering the differences in controlled vocabulary and syntax rules. No language or publication date restrictions were applied.

### Electronic search

The search strategies for 4 major databases are shown in Appendix 1. On June 11, 2021, we searched the following electronic databases to find reports of relevant published studies:

- The Cochrane Central Register of Controlled Trials (CENTRAL);
- MEDLINE (PubMed);
- MEDLINE through OVID (In-Process & Other Non-Indexed Citations);
- EMBASE through OVID;
- LILACS

### Unpublished literature search

In order to further identify potential articles for inclusion, grey literature was searched in the register of clinical studies hosted by the US National Institutes of Health (www.clinicaltrials.gov), the multidisciplinary European database (www.opengrey.eu), the National Research Register, and Pro-Quest Dissertation Abstracts and Thesis databases (https://about.proquest.com).

### Manual search

The reference lists of all identified eligible studies and other published systematic reviews were hand-searched in order to identify further eligible studies.

### Study selection

Study selection was performed independently and in duplicate by the first 2 authors (A.R., J-C.I.) of the review, who were not blinded to the identity of the authors of the studies, their institutions, or the results of their research. Calibration among the reviewers was performed in the first 20 studies retrieved.

Study selection procedure comprised of title- and abstract-reading followed by full-text-reading stages. After exclusion of non-eligible studies, the full report of publications considered by either author as eligible for inclusion was obtained and assessed independently. In studies reporting on the same patient sample, only the publication with the longest follow-up was considered. Disagreements were resolved by discussion and consultation with the third author of the review (A.St.). Inter-observer agreement was assessed for the screening of full-text articles. A record of all decisions on study identification was kept.

### Data collection

The first and third authors (A.R., A.St.) performed data extraction independently and in duplicate. Disagreements were resolved by discussion with the latest two authors.

Specifically designed excel collection forms were used to record the desired information.

The following data were collected: author/title/year of study, study affiliation data, design of the study, number/age/gender/smoking status of participants in intervention and control groups, intervention applied, and outcome assessed with all relevant clinical and radiographic variables.

If stated, the sources of funding, trial registration, and publishing of the trial’s protocol were recorded. This information was used to aid assessment of heterogeneity and the external validity of the included studies.

In case of missing data, it was attempted to contact the corresponding author. Studies without enough data for meta-analyses were kept in the systematic review but excluded from the meta-analyses.

### Risk of bias assessment

Risk of Bias 2.0 tool (RoB) was used to assess the quality of the included randomized studies [33]. Risk of bias assessment was performed independently and in duplicate by the first and third authors (A.R., A.St.) for the primary outcomes. Any concern was resolved by discussion with the last two authors.

### Data analysis

Meta-analyses were conducted with included studies reporting similar interventions and comparable outcomes in homogeneous samples, i.e., in the case of limited heterogeneity. For continuous variables, mean differences, and standard deviations were used to summarize the data from each study. Mean differences and 95% CIs were calculated across studies. Data were analyzed with Review Manager 5.4 (Review Manager (RevMan), Version 5.4, The Cochrane Collaboration, Copenhagen, 2020).

### Heterogeneity

Clinical and methodological heterogeneity were assessed by examining the characteristics of the studies, the similarity between the types of participants, the interventions, and the outcomes as specified in the inclusion criteria for considering studies for this review. Statistical heterogeneity was assessed using a chi^2^ test and the *I*^2^ statistic.

### Subgroup analyses

In the case of sufficient data, subgroup analyses to explore the influence of study characteristics such as age, gender, and/or jaw were planned to be conducted.

### Sensitivity analysis

We explored whether or not the analysis of studies stratified by design or by risk of bias (i.e., overall low risk versus high risk) yielded similar or different results.

### Unit of analysis issues

We anticipated that some of the included studies presented data from repeated or paired observations on participants, which could lead to unit of analysis errors. In such cases, we followed the advice provided in Section 9.3.4 of the Cochrane Handbook for Systematic Reviews of Interventions [33].

### Assessment of reporting bias

Potential reporting biases including publication bias, multiple (duplicate reports) publication bias, and language bias in this review were reduced by conducting an accurate and at the same time a sensitive search of multiple sources with no restriction on language. A search for ongoing trials was conducted too. In the presence of more than 10 studies in a meta-analysis, the possible presence of publication bias would have been investigated for the primary outcome.

## Results

### Search

**Fig. 1:**
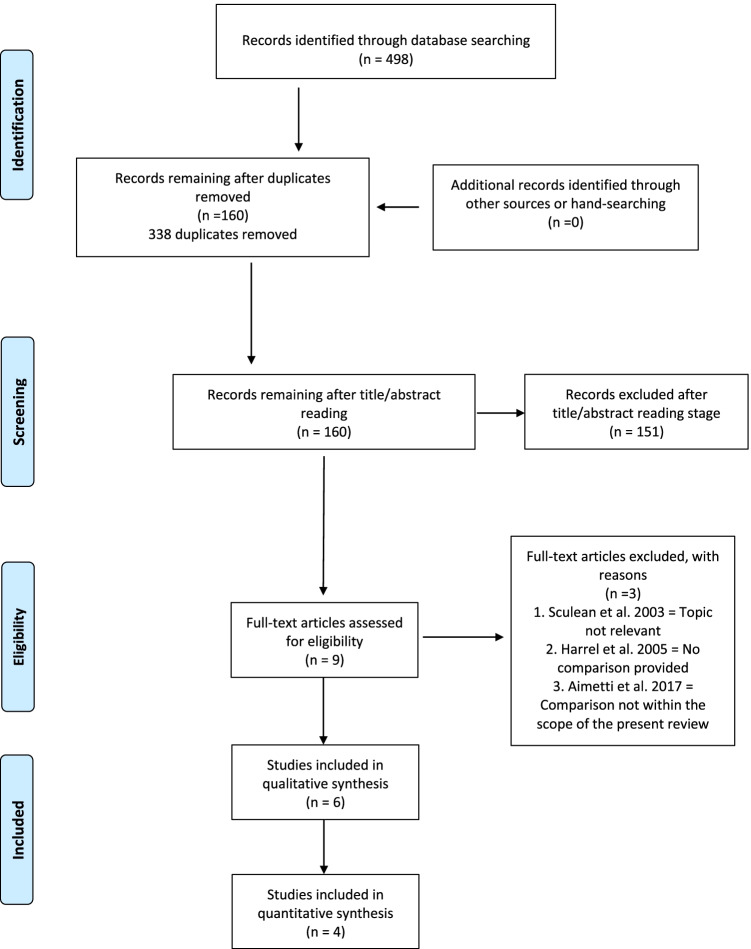
Study flow-chart

Through electronic search, a total of 607 records were identified. After duplicate removal, 160 records underwent abstract screening. No publications were retrieved from the manual searches. Upon exclusion of one-hundred and fifty-one articles based on their abstracts, nine articles remained for full-text evaluation. After exclusion of three articles [34–36] based on full-text analysis, 6 articles [25, 27–29, 37, 38] remained for qualitative (*n* = 6) and quantitative analysis (*n* = 4). Calibration among authors indicated complete agreement (i.e., *k*-score = 1, agreement = 100%) for the study selection process. Details of the search process are provided in Fig. 1.

### Characteristics of included studies

#### Study design

Two studies were conducted as multicenter [28, 29] and 4 as single-center trials [25, 27, 37, 38]. Five articles were split-mouth RCTs [25, 28, 29, 37, 38] while 1 study was a parallel-arm RCT [27].

#### Studies’ samples

Sample sizes varied from 16 to 49 patients. A power calculation was reported in 2 RCTs [27, 28]. The age of the patients in all included studies ranged from 18 to 85 years. All studies, except 2, reported on gender distribution [37, 38]. All studies included smoking patients, except one study [38].

#### Intervention/Comparison

Details of the interventions are described in Table 1. Briefly, all the included studies clearly reported on the NSPT performed under local anesthesia whether with hand and/or ultrasonic instrumentation. Five studies [25, 27, 29, 37, 38] reported on NSPT with or without the use of EMD in non-treated periodontitis patients, while one study [28] showed clinical results after retreatment of selected sites. The application of EMD in test sites was performed always once, expect Schallhorn and co-workers [29] who re-applied EMD after 2 to 3 weeks. One study [37] included 4 groups of which two received NSPT and adjunctive systemic antibiotics (AB) either with or without the use of EMD. Inclusion of test and control sites varied among the studies. One study included all sites ≥ 6 mm in a parallel group design [27]; another study included only one inter-proximal site with an intrabony defect ≥ 2 mm and PPD ≥ 5 mm per patient [37]. Others included two sites per patient, one experimental and one control [25] or at least two teeth with similar PPD ≥ 5 mm and ≤ 8 mm, BoP in a split-mouth design [28]. Two studies included at least 2 pockets per contralateral quadrants, also in a split-mouth design [29, 38].

**Table 1:** Details of the studies’ characteristics

	Author	Study title	Study design	Year	Participants	Interventions	Outcomes	Notes
1	Graziani et al	Enamel matrix derivative stabilizes blood clot and improves clinical healing in deep pockets after flapless periodontal therapy: a randomized clinical trial	RCT, parallel-arm, single center, follow-up: 3 months	2019	Number of patients: 38, no drop-outs Sex: 47.4% females in test group; 57.9% females in control group Age (years, mean): 50.11 ± 8.92 in test group; 50.89 ± 10.08 in the control group Smoking: 22.2% in test group; 47.4% in control group Inclusion criteria: (1) proximal attachment loss of ≥ 3 mm in ≥ 2 non-adjacent teeth, (2) bleeding on probing on at least 25% of total sites, and (3) documented radiographic bone loss Included sites: each site with ≥ 6 mm Exclusion criteria: (1) younger than 18 years and older than 75 years of age, (2) pregnant or lactating females, (3) females using hormonal contraceptive methods, (4) reported diagnosis of any systemic illnesses including cardiovascular, renal, and liver diseases, (5) any pharmacological treatment within the 3 months before the beginning of the study, (6) smoking more than 20 cigarettes/day, and (7) periodontal treatment in the previous 6 months	Initial periodontal therapy: NR Treatment at test sites (SRP + EMD): periodontal treatment, consisting of both supra- and subgingival mechanical instrumentation of the root surface (debridement and scaling) with ultrasonic instrumentation with fine tips only (EMS; Nyon, Switzerland). Treatment was delivered within 1 h exactly in all cases according to the method “Full-mouth ultrasonic debridement.” At the completion of the treatment, the operator left the clinical room and another clinician applied 24% EDTA (Prefgel®; Institut Straumann AG) with a sterile syringe with a thin blunt tip (25GX1/4ʺ) in each site with PPD ≥ 6 mm. The sites were then copiously rinsed with both water-spray and by 5-s passage of ultrasonic instrument’s fine tip in the site with no contact to the root surface. After irrigation, sterile gauze was placed in the vestibule in the proximity of a selected site and a thorough drying of the site was performed with an air-spray. Afterwards, an orthodontic floss (Superfloss®; Oral B, Ireland) was placed and left in the site for 1 min. Irrigation and floss application were repeated until complete bleeding control. Once bleeding control was achieved, EMD (Straumann AG) was applied with another blunt tipped sterile syringe (25GX1/4ʺ), until overflowing from the pocket border Treatment at control sites (SRP): periodontal treatment, consisting of both supra- and subgingival mechanical instrumentation of the root surface (debridement and scaling) with ultrasonic instrumentation with fine tips only (EMS; Nyon, Switzerland). Treatment was delivered within 1 h exactly in all cases according to the method “Full-mouth ultrasonic debridement.” Supportive periodontal therapy: OHI sessions after 1 day, 1 week, and each month after treatment, until completion of the study	CAL: Mean CAL change test (baseline–3 months, mm): from 4.2 ± 0.81 to 3.24 ± 0.93 Mean CAL change control (baseline–3 months, mm): from 4.18 ± 0.78 to 3.43 ± 1.06 Mean CAL in sites ≥ 6 mm test (baseline–3 months, mm): from7.31 ± 0.93 to 4.40 ± 1.38 Mean CAL in sites ≥ 6 mm control (baseline–3 months, mm): from 7.04 ± 0.68 to 5.11 ± 1.29 PPD: PPD change test (baseline–3 months, mm): from 3.85 ± 0.56 to 2.72 ± 0.59 PPD change control (baseline–3 months, mm): from 3.96 ± 0.67 to 3.02 ± 0.69 Mean PPD in sites ≥ 6 mm test (baseline–3 months, mm): from 7.06 ± 0.61 to 3.81 ± 1.08* Mean PPD in sites ≥ 6 mm control (baseline–3 months, mm): from 6.81 ± 0.54 to 4.70 ± 1.01 % of sites PPD ≥ 6 mm test (baseline–3 months, mm): from 14.46 ± 10.48 to 3.25 ± 4.95* % of sites PPD ≥ 6 mm control (baseline–3 months, mm): from 17.7 ± 12.85 to 7.09 ± 8.03	Funding: Straumann AG, Switzerland Sample size calculation: Yes Registration: Yes
2	Gutierrez et al	Evaluation of enamel matrix derivative as an adjunct to non-surgical periodontal therapy	RCT, split-mouth, single-center, follow-up: 3 months	2003	Number of patients: 22, 2 drop-outs Sex: 11 males, 11 females; drop-outs: 1 male, 1 female Age (years): NR Smoking: 10 patients Inclusion criteria: (1) age > 18 years, (2) diagnosis of chronic periodontitis, (3) no subgingival scaling within the past 6 months Included sites: two non-adjacent sites with pockets ≥ 5 mm associated with single rooted teeth, and (5) with radiographic angular bone defects > 3 mm. (5) Two sites around teeth with similar anatomy and associated with similar intrabony defect morphology were chosen in each patient Exclusion criteria: (1) Subjects included in other clinical trials involving therapeutic intervention, (2) experimental teeth that did not respond positively to pulp testing, (3) uncontrolled systemic illnesses (i.e., diabetes mellitus, cancer, HIV, bone metabolic diseases, or disorders that compromise wound healing, chronic high-dose steroid therapy, radiation, or immune-suppressive therapy), (4) subjects with acute infectious lesions in the experimental areas, and (5) subjects having received systemic antibiotic treatment within the previous 2 months	Initial periodontal therapy: OHI Treatment test sites (SRP + EMD):SRP under local anesthetic in a single appointment. SRP (by hand and/or ultrasonic instruments) was performed to the depth of the periodontal pocket until the operator felt that the root surface was hard and smooth. No time restraint was placed on the scaling procedure (mean duration B5–10 min/tooth), rinsed with saline solution, followed by manual compression of gingival tissues until no persistent bleeding could be detected. Using a subgingival irrigation syringe, a 24% EDTA gel (Prefgel, BIORA AB, Malmo, Sweden) was delivered into both experimental and control sites. After 2 min, the pockets were again irrigated with sterile saline. Experimental sites were then treated with enamel matrix derivative, 30 mg/ml (Emdogain, BIORA AB, Malmo, Sweden); the EMD gel was delivered subgingivally using a blunt 22-gauge needle placed at the bottom of the pocket. The gel was applied until the pocket was overfilled. Pressure with moist gauze was applied to the site for 5 min following delivery of the gel Treatment control sites (SRP): SRP under local anesthetic in a single appointment. SRP (by hand and/or ultrasonic instruments) was performed to the depth of the periodontal pocket until the operator felt that the root surface was hard and smooth. No time restraint was placed on the scaling procedure (mean duration B5–10 min/tooth).OFD. rinsed with saline solution, followed by manual compression of gingival tissues until no persistent bleeding could be detected. Using a subgingival irrigation syringe, a 24% EDTA gel (Prefgel, BIORA AB, Malmo, Sweden) was delivered into both experimental and control sites. After 2 min, the pockets were again irrigated with sterile saline Supportive periodontal therapy: 1 month post-treatment, supragingival prophylaxis and OHI	CAL: CAL change test after 3 months: 1.4 ± 0.3 mm CAL change control after 3 months: 1.8 ± 0.4 mm PPD: PPD change test after 3 months: 2.0 ± 0.3 mm; PPD change control after 3 months: 2.3 ± 0.5 mm BoP: BoP test after 3 months: 40% BoP control after 3 months: 30% PI: PI test after 3 months: 45% PI control after 3 months: 40%	Funding: NR Sample size calculation: NR Registration: NR
3	Jentsch et al	Flapless application of enamel matrix derivative in periodontal retreatment: a multicentre randomized feasibility trial in periodontal patients with anteriorly displaced incisors	RCT, split-mouth, multicenter, Follow-up: 12 months	2021	Number of patients: 44, 1 drop-out after 6 months: 43 patients, 3 additional drop-outs after 12 months: 40 patients Sex: 21 males, 23 females Age (years, range): 31–74 Smoking: 11 Inclusion criteria: (1) Stage III periodontitis, (2) at reevaluation (3 to 6 months after initial therapy) Included sites: at least 2 residual pockets with PPD ≥ 5 and ≤ 8 mm, BoP positive, mobility ≤ degree 1 without furcation involvement, experimental teeth with similar PPD had to be located in different quadrants or at least 3 teeth apart from each other Exclusion criteria: (1) full-mouth plaque score > 20%, (2) uncontrolled systemic disease, (3) requiring high-dose steroids, (4) radiation or other immune-suppressive therapy, (5) history of malignant disease in the oral cavity or previous radiotherapy in the head or neck area, (6) pregnant or lactating females, (7) drug and alcohol abuse, (8) smoking > 10 cigarettes/day, (9) inadequate restorative therapy or malocclusion	Initial periodontal therapy: subgingival SRP with hand and/or power-driven instruments under local anesthesia, oral hygiene instructions, reevaluation after 3 or 6 months Treatment at test sites (SRP + EMD): retreatment of selected sites. In local anesthesia, SRP with mini curettes (Hu-Friedy) and ultrasonic instruments with thin and delicate tips (Perio Slim, EMS). Root conditioning for 2 min with EDTA (PrefGel, Institut Straumann AG), rinsed with saline solution and thoroughly dried. Blood removed with paper points, gauze swabs, and/or sponge pellets followed by repeated irrigation and air-drying until complete bleeding control. EMD application (EMD, Institut Straumann AG) until overflowing from the gingival margin. With sterile wetting gauzes, the gingival margin was compressed pocket closure was obtained Treatment control sites (SRP): retreatment of selected sites. In local anesthesia, SRP with mini curettes (Hu-Friedy) and ultrasonic instruments with thin and delicate tips (Perio Slim, EMS) Supportive periodontal therapy: OHI and supragingival plaque removal every week for the first month, thereafter every 3 months	CAL: NR PPD: PPD change test (baseline–6 months, mm): from 6.0 ± 0.9 to 3.9 ± 1.2 PPD change control (baseline–6 months, mm): from 5.9 ± 0.9 mm to 4.6 ± 1.2 mm PPD test (mm) at 12 months: 3.9 ± 1.2 mm PPD control (mm) at 12 months: 4.6 ± 1.1 mm BoP: BoP test at 6 months: 9.3%* BoP control at 6 months: 27.9% BoP test at 12 months: 5.0%* BoP control at 12 months: 22.5% Conversion of deep sites into shallow sites: Frequency of conversion of residual deep sites to PPD ≤ 4 mm (irrespective of BoP): test: 6 months:76%*; 12 months:80%*; control: 6 months:46%; 12 months:45% Frequency of conversion of pocket closure to PPD ≤ 4 mm, no BoP: test: 6 months:69%*; 12 months:80%*; control: 6 months:34%; 12 months:42%:	Funding: Straumann AG, Switzerland Sample size calculation: Yes Registration: Yes
4	Mombelli et al	Enamel matrix proteins and systemic antibiotics as adjuncts to non-surgical periodontal treatment: clinical effects	RCT, split-mouth, single-center, follow-up: 12 months	2005	Number of patients: 16, no drop-outs Sex: NR Age (years): NR Smoking: smoking history was recorded, number of smokers: NR Inclusion criteria: (1) age 25–65 years, (2) presence of inter-proximal periodontal lesions in each of two contralateral quadrants in the region including the canine, premolars, and the mesial aspect of the first molar, (3) presence of Porphyromonas gingivalis in a pooled subgingival plaque sample from this region, Included sites: one inter-proximal periodontal lesion with radiographic evidence of an intrabony defect ≥ 2 mm in depth, associated with a pocket probing depth (PPD) ≥ 5 mm, and clinical attachment level (CAL) ≥ 5 mm and bleeding on controlled probing force Exclusion criteria: (1) Enrollment in another clinical trial (either medical or dental), (2) systemic illnesses (i.e., diabetes mellitus, cancer, HIV, bone metabolic diseases, or disorders that compromise wound healing, chronic high-dose steroid therapy, radiation, or immune-suppressive therapy), (3) pregnancy or lactation, (4) systemic antibiotics taken within the previous 2 months, (5) confirmed or suspected intolerance to 5-nitroimidazole- derivatives or amoxicillin, and (6) subgingival scaling in the last year	Initial periodontal therapy: OHI; Excluding the selected study sites (one test, one control), all teeth with ≥ 4 mm PPD were thoroughly scaled and root planed with ultrasonic and hand instruments. Treatment was continued until the operator felt that all tooth surfaces were clean, hard, and smooth. This was accomplished in two to four treatment sessions, scheduled 1 week apart Treatment at test sites (SRP + EMD): In a single, separate session: Thorough SRP, followed by pocket irrigation with saline solution, manual compression of gingival tissues for 5 min, and the placement of a retraction chord containing 10% potassium sulfate (GingiBraid 3a, Van R Dental Products, Oxnard, CA, USA). The retraction chords were removed after 2 min in place, and then, the sites were rinsed with saline. PrefGel was applied in the pockets during 2 min, followed by another saline irrigation. With a blunt cannula inserted to the bottom of the pocket. Sterile lyophilized EMD (Emdogain, Biora AB, Malm, Sweden), was applied until spill over Treatment at control sites (SRP + placebo): Fixed appliances, segmented arch technique, intrusive arches and cantilevers using light and continuous forces about 10 to 15 g, posterior anchorage obtained by means of palatal arches and stainless steel segments. 6 adults received orthognathic surgery Additional treatment modality with or without AB: Group 1 (AB): At the end of the session, the subjects received a neutral package containing metronidazole and amoxicillin to take of each tablet three times a day for 7 consecutive days Group 2 (placebo): At the end of the session, the subjects received a neutral package containing placebo, to take of each tablet three times a day for 7 consecutive days Supportive periodontal therapy: After 10 days, 2, 6, and 12 months, OHI and supragingival calculus removal	CAL: Mean CAL change test (baseline–6 months, mm): from 8.6 ± 2.9 to 6.6 ± 2.3 Mean CAL change control (baseline–6 months, mm): from 7.6 ± 2.0 to 6.7 ± 2.0 Mean CAL change test (baseline–12 months, mm): from 8.6 ± 2.9 to 6.1 ± 3.4 Mean CAL change control (baseline–12 months, mm): from 7.6 ± 2.0 to 5.8 ± 2.5 PPD: Mean PPD change test (baseline–6 months, mm): from 7.6 ± 1.3 to 4.8 ± 1.7 Mean PPD change control (baseline–6 months, mm): from 6.9 ± 1.7 to 5.1 ± 1.5 Mean PPD change test (baseline–12 months, mm): from 7.6 ± 1.3 mm to 5.4 ± 1.9 mm Mean PPD change control (baseline–12 months, mm): from 6.9 ± 1.7 to 4.9 ± 1.4 BoP: Only reported for comparison between treatment with or without AB	Funding: Biora Malmö, Sweden Sample size calculation: NR Registration: NR
5	Schallhorn et al	Application of enamel matrix derivative in conjunction with non-surgical therapy for treatment of moderate to severe periodontitis: a 12-month, randomized prospective, multicenter study	RCT, split-mouth, multicenter, follow-up: 12 months	2019	Number of patients: 49, 4 drop-outs Sex: 51% female and 49% male Age (years): mean: 55.2 ± 11.3, range: 31–85 Smoking: 12 patients were smokers Inclusion criteria: (1) 18–85 years of age, (2) no contraindications to periodontal therapy Included sites: at least two pockets per contralateral quadrants within one arch with PPD 5–8 mm Exclusion criteria: (1) patients unable or unwilling to provide informed consent, (2) uncontrolled systemic diseases, chronic high-dose steroid therapy, bone metabolic disease, radiation, or immuno-suppressive therapy, infections at treatment sites, (3) heavy smoking (> 10 cigarettes per day or > 1 cigar per day) or smokeless tobacco use, (4) drug addiction or alcohol abuse, (5) current systemic antibiotic treatment or within 3 months prior to the study, (6) SRP or periodontal surgery within 6 months, (7) pregnancy, (8) necrotizing ulcerative periodontitis or periodontitis as a manifestation of systemic disease, (9) teeth with probing pocket depths ≥ 9 mm, furcation involvement, and/or mobility degree > 1, (10) test and control sites on adjacent teeth in the two-quadrants	Initial periodontal therapy: Treatment at test sites (SRP + EMD): Patients received local anesthetic and were treated in a single visit. For the quadrant assigned to SRP with EMD, the following steps were conducted: SRP, control of bleeding using manual pressure or gauze, EDTA application (until overflow from pocket was observed) for 2 min, sterile water irrigation, application of EMD (utilizing manufacturer provided cannula, Straumann AG) until overflow from the pocket was observed. 2–3 weeks after visit 2, EMD was re-applied to the test quadrant. The procedure involved no anesthesia, supragingival plaque removal utilizing hand instruments, no EDTA application, and application of EMD starting apically and advancing coronally Treatment at control sites (SRP): Patients received local anesthetic and were treated in a single visit Supportive periodontal therapy: 3, 6, 9, and 12 months, supra- and subgingival hand and ultrasonic scaling	CAL: CAL change test after 12 months (mm): − 2.2 ± 1.5 CAL change control after 12 months (mm): − 2.1 ± 1.3 PPD: PPD change test after 12 months (mm): − 2.4 ± 1.3 PPD change control after 12 months (mm): − 2.3 ± 1.2 PPD change for the 5–8 mm subgroup test after 12 months (mm): − 2.0 ± 0.7 PPD change for the 5–8 mm subgroup control after 12 months (mm): − 1.4 ± 0.8 % of healthy PPD < 5 mm test at 12 months: 89.2 % of healthy PPD < 5 mm control at 12 months: 80.6 BOP: BOP test at 12 months (%):17.8* BOP control at 12 months (%):23.3 Pocket conversion: Pockets converted to no longer requiring surgical intervention (< 5 mm) test after 12 months: 79.8%* Pockets converted to no longer requiring surgical intervention (< 5 mm) control after 12 months: 65.9% Recession: Change in gingival margin (mm) test after 12 months: − 0.3 ± 0.9 Change in gingival margin (mm) control after 12 months: − 0.2 ± 1.0	Funding: Straumann AG, Switzerland Sample size calculation: NR Registration: Yes
6	Wyganowska et al	The evaluation of enamel matrix derivative on subgingival microbial environment in non-surgical periodontal therapy	RCT, split-mouth, single-center, follow-up: 3 months	2013	Number of patients: 20, no drop-outs Sex: NR Age (years, mean): 42.3 Smoking: no smokers included Inclusion criteria: (1) clinically diagnosed medium or severe chronic periodontitis (PPD ≥ 5 mm and CAL ≥ 3 mm) Included sites: two upper quadrants with comparable clinical states (at least two pockets with PPD ≥ 6 mm) Exclusion criteria: (1) periodontal disease treatment in the last six months, (2) concomitant general diseases, (3) antibiotic therapy in the last 3 months, and (4) smoking	Initial periodontal therapy: NR Treatment at test sites (SRP + EMD): SRP was carried out immediately in the whole oral cavity by means of manual and ultrasound tools, avoiding the use of antiseptics both during the time preceding the Emdogain application and afterwards (FMSRP) Two days after the therapy, Emdogain preparation (Straumann, Basel, Switzerland) from Schlamberger (Warsaw, Poland) was applied into the pockets in one quadrant. The application of the drug was preceded by inserting PrefGel preparation into the pockets for two minutes. The preparation was thoroughly washed away. applying subgingival irrigation (Perio Pic) with the use of saline Treatment at control sites (SRP): SRP was carried out immediately in the whole oral cavity by means of manual and ultrasound tools, avoiding the use of antiseptics both during the time preceding the Emdogain application and afterwards (FMSRP) Supportive periodontal therapy: NR	CAL: Mean CAL change test (baseline–3 months, mm): from 5.2 ± 1.4 to ± 3.85 ± 0.93 Mean CAL change control (baseline–3 months, mm): from 5.3 ± 1.26 to 4.0 ± 0.86 PPD: Mean PPD change test (baseline–3 months, mm): from 7.00 ± 1.81 to 4.10 ± 0.97 Mean PPD change control (baseline–3 months, mm): from 6.95 ± 1.88 to 4.30 ± 1.08 BOP: BOP change test at 3 months: 44.75% BOP change control at 3 months:42.5% PI: PI change test at 3 months: 46.25% PI change control at 3 months:45.75%	Funding: NR Sample size calculation: NR Registration: NR

### Outcomes

#### Primary outcome

All studies, except Jentsch et al. [28], reported on CAL changes. Detailed information regarding CAL changes are summarized in Table 2. CAL change among the studies ranged from 0.75 to 2.91 mm. Although all studies reported higher values for CAL change for the test compared to the control groups, none of the studies reported on a significant difference among test and control groups. Graziani and co-workers [27] were the only ones distinguishing between sites ≤ 5 mm and ≥ 6 mm. Of the two papers [29, 37] that presented CAL change after a follow-up time of 12 months, only one [37] presented 6-month and 12-month results demonstrating a stable CAL after NSPT with no further changes from the 6-month to the 12-month follow-up. Mombelli and co-workers [37] included additional two groups receiving systemic antibiotics or a placebo. No statistical difference was discerned when the groups with or without EMD were compared with each other irrespective of the antibiotic therapy they received (i.e., groups NSPT + AB and NSPT vs NSPT + AB + EMD and NSPT + EMD). However, the sites treated with both EMD and AB showed a synergistic effect gaining the largest amount of CAL and when comparing this group with the other three a significant difference favoring AB + EMD was found for all 3 time points that were assessed (i.e., 2, 6, and 12 months).

**Table 2:** Risk of bias assessment

	Author/ Year	Study title	Bias arising from the randomization process	Bias due to deviations from the intended interventions	Bias due to missing outcome data	Bias in measurement of the outcome	Bias in selection of the reported result	Overall bias
1	Graziani et al. 2019 [27]	Enamel matrix derivative stabilizes blood clot and improves clinical healing in deep pockets after flapless periodontal therapy: a randomized clinical trial	Authors’ judgment: Low risk Support for judgment: randomization process and allocation are in detail explained	Authors’ judgment: Low risk? Support for judgment: protocol straight forward no clue of deviation	Authors’ judgment: Low risk Support for judgment: all outcome data available	Authors’ judgment: Low risk Support for judgment: not clear whether outcome assessors blinded	Authors’ judgment: Low risk Support for judgment: reported outcome data unlikely to have been selected	Authors’ judgment: Low risk
2	Gutierrez et al. 2003 [25]	Evaluation of enamel matrix derivative as an adjunct to non-surgical periodontal therapy	Authors’ judgment: Low risk Support for judgment: details about randomization process and allocation	Authors’ judgment: Low risk Support for judgment: protocol straight forward no clue of deviation	Authors’ judgment: Low risk Support for judgment: all outcome data available	Authors’ judgment: Low risk Support for judgment: blinded outcome assessor	Authors’ judgment: Low risk Support for judgment: reported outcome data unlikely to have been selected	Authors’ judgment: Low risk
3	Mombelli et al. 2005 [37]	Enamel matrix proteins and systemic antibiotics as adjuncts to non-surgical periodontal treatment: clinical effects	Authors’ judgment: Low risk Support for judgment: details about randomization process and allocation	Authors’ judgment: Low risk Support for judgment: protocol straight forward no clue of deviation	Authors’ judgment: Low risk Support for judgment: all outcome data available	Authors’ judgment: Low risk Support for judgment: clearly stated who performed the treatment and who the outcome assessments	Authors’ judgment: Low risk? Support for judgment: reported outcome data unlikely to have been selected	Authors’ judgment: Unclear risk
4	Jentsch et al. 2021 [28]	Flapless application of enamel matrix derivative in periodontal retreatment: a multicentre randomized feasibility	Authors’ judgment: Low risk Support for judgment: randomization conducted by a person not involved in the study by tossing a coin	Authors’ judgment: Low risk Support for judgment: protocol straight forward no clue of deviation	Authors’ judgment: Low risk Support for judgment: all outcome data available	Authors’ judgment: Low risk Support for judgment: calibrated and blinded examiner not involved in the treatment	Authors’ judgment: Low risk Support for judgment: reported outcome data unlikely to have been selected	Authors’ judgment: Low risk
5	Schallhorn et al. 2021 [29]	Application of enamel matrix derivative in conjunction with non-surgical therapy for treatment of moderate to severe periodontitis: a 12-month, randomized prospective, multicenter study	Authors’ judgment: Unclear risk Support for judgment: no information about randomization process	Authors’ judgment: Low risk Support for judgment: protocol straight forward no clue of deviation	Authors’ judgment: Low risk Support for judgment: all outcome data available	Authors’ judgment: Unclear risk Support for judgment: not mentioned whether outcome assessor was blinded or not	Authors’ judgment: Low risk Support for judgment: reported outcome data unlikely to have been selected	Authors’ judgment: Unclear risk
6	Wyganowska et al. 2013 [38]	The evaluation of enamel matrix derivative on subgingival microbial environment in non-surgical periodontal therapy	Authors’ judgment: Unclear Support for judgment: not clear how randomization was performed	Authors’ judgment: Low risk Support for judgment: no clue of deviation	Authors’ judgment: Low risk Support for judgment: all outcome data available	Authors’ judgment: Unclear risk Support for judgment: no information about outcome assessors	Authors’ judgment: Low risk Support for judgment: reported outcome data unlikely to have been selected	Authors’ judgment: Low risk

#### Secondary outcomes

All studies reported on the secondary outcome PPD change. Details are reported in Table 2. While 4 studies reported on average PPD change for all sites, two studies distinguished between ≤ 4 mm or ≤ 5 mm and ≥ 6 mm [27, 29]. Sites > 6 mm demonstrated a significantly better result when EMD was used (Graziani et al. [27]). For mean PPD change, Jentsch et al. [28] observed an additional benefit of 0.79 ± 1.3 mm for the test group compared to the control. Two studies evaluated the frequency of pocket closure [28, 29] reporting on significantly better results for test groups. BoP was determined by four studies of which 2 observed a significant difference favoring test conditions [28, 29].

### Meta-analysis

**Fig. 2:**
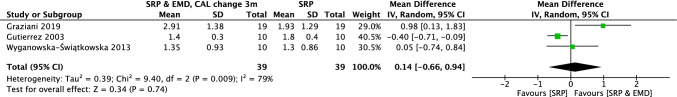
Forest plot with respect to the primary (i.e., CAL change)

**Fig. 3:**
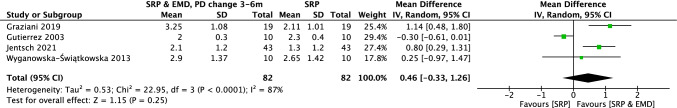
Forest plot with respect to the secondary outcome (i.e., PPD change)

Meta-analysis could only be performed for CAL change at a 3-month follow-up, with data collected from 3 included studies [25, 27, 38] and for PPD change at a 3–6-month follow-up with data from 4 studies [25, 27, 28, 38]. Random effects model was implemented. The meta-analysis revealed only a trend in favor of SRP + EMD versus SRP alone: a non-statistically significant CAL gain of 0.14 mm (*p* = 0.74; CI 95% − 0.66; 0.94) in favor of SRP + EMD compared to SRP alone was discovered. With respect to PPD, again, a non-statistically significant additional PPD reduction of 0.46 mm (*p* = 0.25; CI 95% − 0.33; 1.26) in favor of SRP + EMD compared to SRP alone was identified. Statistical heterogeneity was high in both cases (*I*^2^ = 79% and 87%, respectively). None of the other investigated outcomes as well as the CAL and PPD changes at additional time points (i.e., 6–12 months) could be evaluated due to methodological and clinical heterogeneity. Details of the forest plots with respect to the primary (i.e., CAL change) and secondary outcome (i.e., PPD change) are reported in Figs. 2 and 3.

### Risk of bias assessment

Detailed risk of bias assessment is demonstrated in Table 2. Briefly, 4 studies demonstrated a low risk of bias [25, 27, 28, 38] while 2 studies were at unclear risk of bias [29, 37].

## Discussion

The present systematic review has assessed the level of evidence on the potential added benefit of the adjunctive use of EMD in NSPT compared to NSPT alone. The present study is, to our knowledge, the first systematic review on this controversially discussed topic. More specifically, based on the inclusion criteria, 6 RCTs with at least 3-month follow-up were retrieved and data from 4 of them could be mathematically combined. When focusing on the obtained results, minimal changes in terms of CAL change (3 studies) (i.e., 0.14 mm) and PPD reduction (4 studies) (i.e., 0.46 mm) were noted, failing to detect an additional clinical benefit of EMD application following NSPT.

The main goal of periodontal therapy is to eliminate periodontal pockets through a non-surgical and/or surgical approach [39]. Even though scaling and root planing does still represent the “gold standard,” initial supra- and subgingival instrumentation might leave residual pockets of at least 5 mm [40] which do require further treatment [41]. Consequently, as stated by Sanz-Sanchez and co-workers, root surfaces re-instrumentation might be recommended for shallow pockets (i.e., PPD up to 5 mm), while periodontal surgery should be performed in sites with PPD ≥ 6 mm [41].

Between these two treatment options (i.e., non-surgical re-instrumentation and periodontal surgery), several adjuncts to NSPT have been used to increase the chance of pocket closure (i.e., PPD ≤ 4 mm), such as local delivery of chlorhexidine chip [42], doxycycline [43], and tetracycline [44]; local application of lasers or photodynamic therapy [45, 46]; and hyaluronic acid [47], and to reduce patients’ morbidity. Nevertheless, it must be pointed out that none of the proposed techniques has shown additional beneficial clinical outcomes [48]. One the contrary, EMD application might provide a further treatment option to reduce the need for surgery as clearly shown by Jentsch and co-workers [28] and Schallhorn and co-workers [29]. Teeth with furcation involvement often exhibit residual pockets after NSPT or even experience exacerbation of probing depths in supportive therapy. However, none of the included RCTs reported on the closure of involved furcations. Furthermore, most studies did not report on inclusion of furcation sites.

The application of EMD in conjunction with a surgical access was proven to result in periodontal regeneration (i.e., formation of periodontal ligament, root cementum, and bone) in both animal [49] and human histological studies [18, 19], while its flapless application has shown controversial results. More specifically, histological evidence of periodontal regeneration has been reported on 3 out of 4 examined teeth 6-month post-treatment by Mellonig and co-workers [50], while Sculean and co-workers [36] revealed a healing pattern predominantly characterized by formation of a long junctional epithelium. Irrespective of the histologically healing features, the application of EMD has been shown to have anti-inflammatory and antibacterial effects [51, 52] as well as to promote early wound healing (i.e., neo-angiogenesis, periodontal ligament, and fibroblast cell migration) [53]; all these characteristics might explain the positive short-term results (i.e., 3 weeks up to 3 months) reported by some studies [27, 29].

Several patient-related factors such as smoking status [54], self-performed plaque control [55], and adherence to a SPT regime [56] have been proven to influence the outcomes of NSPT. Smokers respond less favorably to periodontal therapy with less PPD reduction and CAL gain than non-smokers [57, 58]. In the present study, only one article clearly excluded smokers [38]. Most of the studies, however, excluded heavy smokers. Furthermore, most of the studies did not report details on the full-mouth plaque score values which might have had an impact on the clinical outcomes. With respect to the SPT regime, all studies except Wyganowska et al. [38] clearly stated that patients were enrolled in a strict supportive periodontal therapy protocol consisting of oral hygiene measure reinforcement and supra- and subgingival instrumentation, if deemed necessary.

One additional aspect that has to be discussed is related to the 3-D defect morphology associated with deep pockets, which has never been clearly reported in any of the included articles, even though it is well known that deep and narrow infrabony defects do have higher self-regenerative potential than horizontal defects [59]. This aspect is even more important when one considers that the flapless application of EMD in intrabony defects has led to encouraging clinical results [34].

It has been documented that to maximize the efficacy of EMD during periodontal surgery, a careful drying of the planed root surfaced has to be performed before its application [60]: consequently, due to the difficulties to perform an accurate bleeding control through a minimal access to the root surfaces through the periodontal sulcus, the real effect of EMD in this clinical scenario might be difficult to be estimated. Further studies are warranted to investigate the efficacy of non-surgical application of EMD also with respect to costs. Nevertheless, EMD presents a further tool in the toolbox during non-surgical periodontal therapy and in certain cases may prevent surgical therapy and thus may reduce costs.

This study does present some limitations such as the low number of included studies and included patients. Moreover, due to the high heterogeneity of the included studies, a number of additional clinically relevant data such as the BoP values and longer evaluation periods (i.e., 12-month follow-up) were missing.

## Conclusion

Within their limits, the present data have failed to show that the application of EMD as adjunct to NSPT may lead to additional clinical benefits in terms of CAL gain and PPD reduction when compared to NST alone. However, due to the high heterogeneity among the included studies, additional well-designed RCTs need to be performed to evaluate the use of EMD in various clinical scenarios, such as initial periodontal therapy or maintenance.

## Supplementary Information

ESM 1 (DOCX 13.3 kb)

## Data Availability

The data that support the findings of this study are available from the corresponding author upon reasonable request.

## References

[1] Abusleme L, Hoare A (2000). Hong BY and Diaz PI (2021) Microbial signatures of health, gingivitis, and periodontitis. Periodontol.

[2] Hajishengallis G (2000). Chavakis T and Lambris JD (2020) Current understanding of periodontal disease pathogenesis and targets for host-modulation therapy. Periodontol.

[3] Hajishengallis G, Lamont RJ (2000). (2021) Polymicrobial communities in periodontal disease: their quasi-organismal nature and dialogue with the host. Periodontol.

[4] Jakubovics NS, Goodman SD, Mashburn-Warren L, Stafford GP, Cieplik F (2021). The dental plaque biofilm matrix. Periodontol.

[5] Papapanou PN, Sanz M, Buduneli N, Dietrich T, Feres M, Fine DH, Flemmig TF, Garcia R, Giannobile WV, Graziani F, Greenwell H, Herrera D, Kao RT, Kebschull M, Kinane DF, Kirkwood KL, Kocher T, Kornman KS, Kumar PS, Loos BG, Machtei E, Meng H, Mombelli A, Needleman I, Offenbacher S, Seymour GJ, Teles R, Tonetti MS (2018). Periodontitis: consensus report of workgroup 2 of the 2017 World Workshop on the Classification of Periodontal and Peri-Implant Diseases and Conditions. J Clin Periodontol.

[6] Darveau RP, Curtis MA (2000). (2021) Oral biofilms revisited: a novel host tissue of bacteriological origin. Periodontol.

[7] Joseph S, Curtis MA (2000). (2021) Microbial transitions from health to disease. Periodontol.

[8] Suvan J, Leira Y, Moreno Sancho FM, Graziani F, Derks J, Tomasi C (2020). Subgingival instrumentation for treatment of periodontitis. A systematic review. J Clin Periodontol.

[9] Salvi GE, Mischler DC, Schmidlin K, Matuliene G, Pjetursson BE, Brägger U, Lang NP (2014). Risk factors associated with the longevity of multi-rooted teeth. Long-term outcomes after active and supportive periodontal therapy. J Clin Periodontol.

[10] Caffesse RG, Sweeney PL, Smith BA (1986). Scaling and root planing with and without periodontal flap surgery. J Clin Periodontol.

[11] Hathaway-Schrader JD, Novince CM (2000). (2021) Maintaining homeostatic control of periodontal bone tissue. Periodontol.

[12] De Ry SP, Roccuzzo A, Lang NP, Sculean A, Salvi GE (2021). Long-term clinical outcomes of periodontal regeneration with enamel matrix derivative: a retrospective cohort study with a mean follow-up of 10 years. J Periodontol.

[13] Roccuzzo M, Marchese S, Dalmasso P, Roccuzzo A (2018). Periodontal regeneration and orthodontic treatment of severely periodontally compromised teeth: 10-year results of a prospective study. Int J Periodontics Restorative Dent.

[14] Jepsen S, Gennai S, Hirschfeld J, Kalemaj Z, Buti J, Graziani F (2020). Regenerative surgical treatment of furcation defects: a systematic review and Bayesian network meta-analysis of randomized clinical trials. J Clin Periodontol.

[15] Nibali L, Koidou VP, Nieri M, Barbato L, Pagliaro U, Cairo F (2020). Regenerative surgery versus access flap for the treatment of intra-bony periodontal defects: a systematic review and meta-analysis. J Clin Periodontol.

[16] Hammarström L, Heijl L, Gestrelius S (1997). Periodontal regeneration in a buccal dehiscence model in monkeys after application of enamel matrix proteins. J Clin Periodontol.

[17] Miron RJ, Sculean A, Cochran DL, Froum S, Zucchelli G, Nemcovsky C, Donos N, Lyngstadaas SP, Deschner J, Dard M, Stavropoulos A, Zhang Y, Trombelli L, Kasaj A, Shirakata Y, Cortellini P, Tonetti M, Rasperini G, Jepsen S, Bosshardt DD (2016). Twenty years of enamel matrix derivative: the past, the present and the future. J Clin Periodontol.

[18] Heijl L (1997). Periodontal regeneration with enamel matrix derivative in one human experimental defect. A case report. J Clin Periodontol.

[19] Sculean A, Donos N, Windisch P, Brecx M, Gera I, Reich E, Karring T (1999). Healing of human intrabony defects following treatment with enamel matrix proteins or guided tissue regeneration. J Periodontal Res.

[20] Di Tullio M, Femminella B, Pilloni A, Romano L, D’Arcangelo C, De Ninis P, Paolantonio M (2013). Treatment of supra-alveolar-type defects by a simplified papilla preservation technique for access flap surgery with or without enamel matrix proteins. J Periodontol.

[21] Graziani F, Gennai S, Cei S, Ducci F, Discepoli N, Carmignani A, Tonetti M (2014). Does enamel matrix derivative application provide additional clinical benefits in residual periodontal pockets associated with suprabony defects? A systematic review and meta-analysis of randomized clinical trials. J Clin Periodontol.

[22] Iorio-Siciliano V, Blasi A, Stratul SI, Ramaglia L, Octavia V, Salvi GE, Sculean A (2021). Healing of periodontal suprabony defects following treatment with open flap debridement with or without an enamel matrix derivative: a randomized controlled clinical study. Clin Oral Investig.

[23] Jentsch H, Purschwitz R (2008). A clinical study evaluating the treatment of supra-alveolar-type defects with access flap surgery with and without an enamel matrix protein derivative: a pilot study. J Clin Periodontol.

[24] Yilmaz S, Kuru B, Altuna-Kiraç E (2003). Enamel matrix proteins in the treatment of periodontal sites with horizontal type of bone loss. J Clin Periodontol.

[25] Gutierrez MA, Mellonig JT, Cochran DL (2003). Evaluation of enamel matrix derivative as an adjunct to non-surgical periodontal therapy. J Clin Periodontol.

[26] Wennström JL, Lindhe J (2002). Some effects of enamel matrix proteins on wound healing in the dento-gingival region. J Clin Periodontol.

[27] Graziani F, Gennai S, Petrini M, Bettini L, Tonetti M (2019). Enamel matrix derivative stabilizes blood clot and improves clinical healing in deep pockets after flapless periodontal therapy: a randomized clinical trial. J Clin Periodontol.

[28] Jentsch HFR, Roccuzzo M, Pilloni A, Kasaj A, Fimmers R, Jepsen S (2021). Flapless application of enamel matrix derivative in periodontal retreatment: a multicentre randomized feasibility trial. J Clin Periodontol.

[29] Schallhorn RA, McClain PK, Benhamou V, Doobrow JH, Grandin HM, Kasaj A (2021). Application of enamel matrix derivative in conjunction with non-surgical therapy for treatment of moderate to severe periodontitis: a 12-month, randomized prospective, multicenter study. J Periodontol.

[30] Moher D, Shamseer L, Clarke M, Ghersi D, Liberati A, Petticrew M, Shekelle P, Stewart LA (2015). Preferred reporting items for systematic review and meta-analysis protocols (PRISMA-P) 2015 statement. Syst Rev.

[31] Tonetti MS, Greenwell H, Kornman KS (2018). Staging and grading of periodontitis: Framework and proposal of a new classification and case definition. J Periodontol.

[32] Stone PW (2002). Popping the (PICO) question in research and evidence-based practice. Appl Nurs Res.

[33] Higgins J and Green S (2011) Cochrane Handbook for Systematic Reviews of Interventions Version 5.1.0. The Cochrane Collaboration

[34] Aimetti M, Ferrarotti F, Mariani GM, Romano F (2017). A novel flapless approach versus minimally invasive surgery in periodontal regeneration with enamel matrix derivative proteins: a 24-month randomized controlled clinical trial. Clin Oral Investig.

[35] Harrel SK, Wilson TG, Nunn ME (2005). Prospective assessment of the use of enamel matrix proteins with minimally invasive surgery. J Periodontol.

[36] Sculean A, Windisch P, Keglevich T, Gera I (2003). Histologic evaluation of human intrabony defects following non-surgical periodontal therapy with and without application of an enamel matrix protein derivative. J Periodontol.

[37] Mombelli A, Brochut P, Plagnat D, Casagni F, Giannopoulou C (2005). Enamel matrix proteins and systemic antibiotics as adjuncts to non-surgical periodontal treatment: clinical effects. J Clin Periodontol.

[38] Wyganowska-Świątkowska M, Szkaradkiewicz AK, Karpiński TM, Marcinkowski JT (2013). The evaluation of enamel matrix derivative on subgingival microbial environment in non-surgical periodontal therapy. Ann Agric Environ Med.

[39] Sanz M, Herrera D, Kebschull M, Chapple I, Jepsen S, Berglundh T, Sculean A, Tonetti MS, Participants EW, Consultants M (2020). Treatment of stage I-III periodontitis—the EFP S3 level clinical practice guideline. J Clin Periodontol.

[40] Tomasi C, Koutouzis T, Wennström JL (2008). Locally delivered doxycycline as an adjunct to mechanical debridement at retreatment of periodontal pockets. J Periodontol.

[41] Sanz-Sánchez I, Montero E, Citterio F, Romano F, Molina A, Aimetti M (2020). Efficacy of access flap procedures compared to subgingival debridement in the treatment of periodontitis. A systematic review and meta-analysis. J Clin Periodontol.

[42] Machtei EE, Hirsh I, Falah M, Shoshani E, Avramoff A, Penhasi A (2011). Multiple applications of flurbiprofen and chlorhexidine chips in patients with chronic periodontitis: a randomized, double blind, parallel, 2-arms clinical trial. J Clin Periodontol.

[43] Lecio G, Ribeiro FV, Pimentel SP, Reis AA, da Silva RVC, Nociti-Jr F, Moura L, Duek E, Casati M, Casarin RCV (2020). Novel 20% doxycycline-loaded PLGA nanospheres as adjunctive therapy in chronic periodontitis in type-2 diabetics: randomized clinical, immune and microbiological trial. Clin Oral Investig.

[44] Aimetti M, Romano F, Torta I, Cirillo D, Caposio P, Romagnoli R (2004). Debridement and local application of tetracycline-loaded fibres in the management of persistent periodontitis: results after 12 months. J Clin Periodontol.

[45] Lin Z, Strauss FJ, Lang NP, Sculean A, Salvi GE, Stähli A (2021). Efficacy of laser monotherapy or non-surgical mechanical instrumentation in the management of untreated periodontitis patients. A systematic review and meta-analysis. Clin Oral Investig.

[46] Salvi GE, Stähli A, Schmidt JC, Ramseier CA, Sculean A, Walter C (2020). Adjunctive laser or antimicrobial photodynamic therapy to non-surgical mechanical instrumentation in patients with untreated periodontitis: a systematic review and meta-analysis. J Clin Periodontol.

[47] Eliezer M, Imber JC, Sculean A, Pandis N, Teich S (2019). Hyaluronic acid as adjunctive to non-surgical and surgical periodontal therapy: a systematic review and meta-analysis. Clin Oral Investig.

[48] Donos N, Calciolari E, Brusselaers N, Goldoni M, Bostanci N, Belibasakis GN (2020). The adjunctive use of host modulators in non-surgical periodontal therapy. A systematic review of randomized, placebo-controlled clinical studies. J Clin Periodontol.

[49] Sculean A, Donos N, Brecx M, Reich E, Karring T (2000). Treatment of intrabony defects with guided tissue regeneration and enamel-matrix-proteins. An experimental study in monkeys. J Clin Periodontol.

[50] Mellonig JT, Valderrama P, Gregory HJ, Cochran DL (2009). Clinical and histologic evaluation of non-surgical periodontal therapy with enamel matrix derivative: a report of four cases. J Periodontol.

[51] Arweiler NB, Auschill TM, Donos N, Sculean A (2002). Antibacterial effect of an enamel matrix protein derivative on in vivo dental biofilm vitality. Clin Oral Investig.

[52] Newman SA, Coscia SA, Jotwani R, Iacono VJ, Cutler CW (2003). Effects of enamel matrix derivative on Porphyromonas gingivalis. J Periodontol.

[53] Yuan K, Chen CL, Lin MT (2003). Enamel matrix derivative exhibits angiogenic effect in vitro and in a murine model. J Clin Periodontol.

[54] Nociti FH (2000). Casati MZ and Duarte PM (2015) Current perspective of the impact of smoking on the progression and treatment of periodontitis. Periodontol.

[55] Arweiler NB (2000). Auschill TM and Sculean A (2018) Patient self-care of periodontal pocket infections. Periodontol.

[56] Ramseier CA, Nydegger M, Walter C, Fischer G, Sculean A, Lang NP, Salvi GE (2019). Time between recall visits and residual probing depths predict long-term stability in patients enrolled in supportive periodontal therapy. J Clin Periodontol.

[57] Chang J, Meng HW, Lalla E, Lee CT (2021). The impact of smoking on non-surgical periodontal therapy: a systematic review and meta-analysis. J Clin Periodontol.

[58] Preber H, Bergström J (1990). Effect of cigarette smoking on periodontal healing following surgical therapy. J Clin Periodontol.

[59] Tsitoura E, Tucker R, Suvan J, Laurell L, Cortellini P, Tonetti M (2004). Baseline radiographic defect angle of the intrabony defect as a prognostic indicator in regenerative periodontal surgery with enamel matrix derivative. J Clin Periodontol.

[60] Miron RJ, Bosshardt DD, Laugisch O, Katsaros C, Buser D, Sculean A (2012). Enamel matrix protein adsorption to root surfaces in the presence or absence of human blood. J Periodontol.

