# Correction to: Enamel matrix derivative as adjunctive to non‑surgical periodontal therapy: a systematic review and meta‑analysis of randomized controlled trials

**DOI:** 10.1007/s00784-022-04508-8

**Published:** 2022-04-27

**Authors:** Andrea Roccuzzo, Jean‑Claude Imber, Alexandra Stähli, Dimitrios Kloukos, Giovanni E. Salvi, Anton Sculean

**Affiliations:** 1 Department of Periodontology, School of Dental Medicine, University of Bern, Freiburgstrasse 7, CH‑3010 Bern, Switzerland; 2 Robert K. Schenk Laboratory of Oral Histology, School of Dental Medicine, University of Bern, Bern, Switzerland; 3 Department of Orthodontics and Dentofacial Orthopedics, School of Dental Medicine, University of Bern, Bern, Switzerland

**Correction to: Clinical Oral Investigations**

**https://doi.org/10.1007/s00784-022-04474-1**

In the published paper, some of the references under the reference section has been incorrectly edited.

The original article has been corrected.

