# Supplementary material for: Enamel matrix derivative as adjunctive to non-surgical periodontal therapy: a systematic review and meta-analysis of randomized controlled trials
Source: Clin Oral Investig. 2022 Apr 7;26(6):4263–80. doi: 10.1007/s00784-022-04474-1 (PMC9203394; doi:10.1007/s00784-022-04474-1)
Supplement: Supplementary file 1 — (DOCX 13.3 kb) [file 784_2022_4474_MOESM1_ESM.docx]

Supplementary material 1

| enamel matrix derivative OR enamel matrix proteins OR EMD OR amelogenins OR emdogain | ((("dental enamel"[MeSH Terms] OR ("dental"[All Fields] AND "enamel"[All Fields]) OR "dental enamel"[All Fields] OR "enamel"[All Fields] OR "enamels"[All Fields] OR "enamel s"[All Fields] OR "enameled"[All Fields] OR "enameling"[All Fields] OR "enamelling"[All Fields]) AND ("altrenogest"[Supplementary Concept] OR "altrenogest"[All Fields] OR "matrix"[All Fields] OR "matrix s"[All Fields] OR "matrixes"[All Fields] OR "matrixs"[All Fields]) AND ("analogs and derivatives"[MeSH Subheading] OR ("analogs"[All Fields] AND "derivatives"[All Fields]) OR "analogs and derivatives"[All Fields] OR "derivatives"[All Fields] OR "derivable"[All Fields] OR "derivant"[All Fields] OR "derivants"[All Fields] OR "derivate"[All Fields] OR "derivated"[All Fields] OR "derivates"[All Fields] OR "derivation"[All Fields] OR "derivations"[All Fields] OR "derivative"[All Fields] OR "derive"[All Fields] OR "derived"[All Fields] OR "derives"[All Fields] OR "deriving"[All Fields])) OR ("enamel matrix proteins"[Supplementary Concept] OR "enamel matrix proteins"[All Fields]) OR "EMD"[All Fields] OR ("amelogenin"[MeSH Terms] OR "amelogenin"[All Fields] OR "amelogenins"[All Fields]) OR ("enamel matrix proteins"[Supplementary Concept] OR "enamel matrix proteins"[All Fields] OR "emdogain"[All Fields])) AND (review[Filter]) |
| --- | --- |
| intrabony defect OR infrabony defect OR infrabony OR intrabony OR periodontal defect OR vertical defect OR periodontitis OR periodontal disease | (("intrabony"[All Fields] AND ("abnormalities"[MeSH Subheading] OR "abnormalities"[All Fields] OR "defects"[All Fields] OR "defect"[All Fields] OR "defect s"[All Fields] OR "defected"[All Fields] OR "defective"[All Fields] OR "defectively"[All Fields] OR "defectives"[All Fields])) OR ("infrabony"[All Fields] AND ("abnormalities"[MeSH Subheading] OR "abnormalities"[All Fields] OR "defects"[All Fields] OR "defect"[All Fields] OR "defect s"[All Fields] OR "defected"[All Fields] OR "defective"[All Fields] OR "defectively"[All Fields] OR "defectives"[All Fields])) OR "infrabony"[All Fields] OR "intrabony"[All Fields] OR (("periodontal"[All Fields] OR "periodontally"[All Fields] OR "periodontically"[All Fields] OR "periodontics"[MeSH Terms] OR "periodontics"[All Fields] OR "periodontic"[All Fields] OR "periodontitis"[MeSH Terms] OR "periodontitis"[All Fields] OR "periodontitides"[All Fields]) AND ("abnormalities"[MeSH Subheading] OR "abnormalities"[All Fields] OR "defects"[All Fields] OR "defect"[All Fields] OR "defect s"[All Fields] OR "defected"[All Fields] OR "defective"[All Fields] OR "defectively"[All Fields] OR "defectives"[All Fields])) OR (("vertical"[All Fields] OR "verticality"[All Fields] OR "vertically"[All Fields] OR "verticals"[All Fields]) AND ("abnormalities"[MeSH Subheading] OR "abnormalities"[All Fields] OR "defects"[All Fields] OR "defect"[All Fields] OR "defect s"[All Fields] OR "defected"[All Fields] OR "defective"[All Fields] OR "defectively"[All Fields] OR "defectives"[All Fields])) OR ("periodontal"[All Fields] OR "periodontally"[All Fields] OR "periodontically"[All Fields] OR "periodontics"[MeSH Terms] OR "periodontics"[All Fields] OR "periodontic"[All Fields] OR "periodontitis"[MeSH Terms] OR "periodontitis"[All Fields] OR "periodontitides"[All Fields]) OR ("periodontal diseases"[MeSH Terms] OR ("periodontal"[All Fields] AND "diseases"[All Fields]) OR "periodontal diseases"[All Fields] OR ("periodontal"[All Fields] AND "disease"[All Fields]) OR "periodontal disease"[All Fields])) AND (review[Filter]) |
| non-surgical OR non surgical OR scaling OR root planing OR debridement OR conventional periodontal therapy OR periodontal treatment OR cleaning OR flapless | ("non-surgical"[All Fields] OR ("non"[All Fields] AND ("surgical procedures, operative"[MeSH Terms] OR ("surgical"[All Fields] AND "procedures"[All Fields] AND "operative"[All Fields]) OR "operative surgical procedures"[All Fields] OR "surgical"[All Fields] OR "surgically"[All Fields] OR "surgicals"[All Fields])) OR ("scale s"[All Fields] OR "scaled"[All Fields] OR "scaling"[All Fields] OR "scalings"[All Fields] OR "weights and measures"[MeSH Terms] OR ("weights"[All Fields] AND "measures"[All Fields]) OR "weights and measures"[All Fields] OR "scale"[All Fields] OR "scales"[All Fields]) OR ("root planing"[MeSH Terms] OR ("root"[All Fields] AND "planing"[All Fields]) OR "root planing"[All Fields]) OR ("debride"[All Fields] OR "debrided"[All Fields] OR "debridement"[MeSH Terms] OR "debridement"[All Fields] OR "debridements"[All Fields] OR "debrides"[All Fields] OR "debriding"[All Fields] OR "debridment"[All Fields] OR "debridments"[All Fields]) OR (("conventional"[All Fields] OR "conventionals"[All Fields]) AND ("periodontal"[All Fields] OR "periodontally"[All Fields] OR "periodontically"[All Fields] OR "periodontics"[MeSH Terms] OR "periodontics"[All Fields] OR "periodontic"[All Fields] OR "periodontitis"[MeSH Terms] OR "periodontitis"[All Fields] OR "periodontitides"[All Fields]) AND ("therapeutics"[MeSH Terms] OR "therapeutics"[All Fields] OR "therapies"[All Fields] OR "therapy"[MeSH Subheading] OR "therapy"[All Fields] OR "therapy s"[All Fields] OR "therapys"[All Fields])) OR (("periodontal"[All Fields] OR "periodontally"[All Fields] OR "periodontically"[All Fields] OR "periodontics"[MeSH Terms] OR "periodontics"[All Fields] OR "periodontic"[All Fields] OR "periodontitis"[MeSH Terms] OR "periodontitis"[All Fields] OR "periodontitides"[All Fields]) AND ("therapeutics"[MeSH Terms] OR "therapeutics"[All Fields] OR "treatments"[All Fields] OR "therapy"[MeSH Subheading] OR "therapy"[All Fields] OR "treatment"[All Fields] OR "treatment s"[All Fields])) OR ("clean"[All Fields] OR "cleaned"[All Fields] OR "cleaning"[All Fields] OR "cleanings"[All Fields] OR "cleanness"[All Fields] OR "cleans"[All Fields]) OR "flapless"[All Fields]) AND (review[Filter]) |
